# 肺癌标志物识别的蛋白质组分析

**DOI:** 10.3779/j.issn.1009-3419.2012.07.11

**Published:** 2012-07-20

**Authors:** William CS CHO, 燕 丁

**Affiliations:** 1 Department of Clinical Oncology, Queen Elizabeth Hospital, Hong Kong; 2 天津医科大学总医院，天津市肺癌研究所，天津市肺癌转移与肿瘤微环境重点实验室; 3 香港特别行政区 伊利沙伯医院 临床肿瘤科

**Keywords:** 癌症生物标志物, 肺癌, 小鼠模型, 血浆, 蛋白质组, 蛋白质组学

## Abstract

在新近蛋白质组学的发展中，对疾病蛋白质组的综合和深入研究已成为重要议题。已有研究报道了在包括肺癌在内的不同疾病中发现的一些生物标志物，有一些在肺癌诊断和预测中有潜在价值。然而，它们很少作为器官特异性生物标志物以充分比较不同肿瘤类别的模型。本文评价了最近发表的一项在不同基因工程小鼠模型中进行比较蛋白质组的研究，并阐明已发现标志物在人肺癌诊断中的有效性及应用。

**评价**：Taguchi A, Politi K, Pitteri SJ et al. Lung cancer signatures in plasma based on proteome profiling of mouse tumor models. Cancer Cell 20(3), 289-299 (2011).

肺癌是世界上癌症死亡的主要原因。肺癌病变涉及许多基因突变和表观遗传学改变的复杂过程。识别反映启动肿瘤进展通路的分子标志物或有助监测肿瘤状态。

检测并定量蛋白质的蛋白质组技术的改进，为试图对疾病进行深入探索的研究人员创造了新的机遇和挑战。高通量蛋白质组学技术联合高等生物信息学被广泛用于识别基于蛋白质通路和信号级联的癌症分子标志物^[1]^。肺癌特异性的蛋白质组学标志物开始涌现。

在最近发表的一篇文章中，Taguchi等^[2]^对数个小鼠肺肿瘤模型中的血浆蛋白质组与其它几种肿瘤类型的血浆蛋白质组进行了对比，并识别出16种与肺癌相关的蛋白质。在EGF受体（EGFR）突变的肺腺癌模型中发现了一个涉及EGFR的蛋白质网络，并在小细胞肺癌（small cell lung cancer, SCLC）模型中发现了一个与神经内分泌进展明显相关的血浆标志物。随后他们将该发现扩展至肺癌患者。其研究提示深入的定量蛋白质组学或可反映肺肿瘤生物学。本文评价了该发现的意义和影响，并讨论了本领域一些相关的研究。

## 方法及结果

在遗传工程小鼠模型中利用定量蛋白质组学方法，Taguchi等比较了四种肺癌模型（*EGFR*^*L858R*^腺癌、*Kras*^*G12D*^腺癌、*p53*^*lox*/*lox*^ SCLC以及*Rb*_*lox*/^*lox*^_ SCLC）与其它肿瘤类型（乳腺癌、胰腺癌、卵巢癌、结肠癌以及前列腺癌）模型、两种炎症（急性和慢性）模型中的血浆蛋白质的表达谱。研究鉴别出一些可反映肺肿瘤生物学的血浆蛋白质谱，包括13种与肺腺癌特异性相关的蛋白质，以及3种蛋白质在肺癌中表达增加而在非肺肿瘤模型中却未见增加。

编码小鼠肺腺癌血浆中水平升高的四种蛋白质的同源基因（*SFTPB*、*SFTPD*、*NPC2*以及*WFDC2*）与*TITF1*/*NKX2*-*1* mRNA水平呈正相关。其中，在111个非小细胞肺癌（non-small cell lung cancer, NSCLC）肿瘤中*NPC2*的表达与*TITF1*/*NKX2*-1 mRNA明显相关。利用两种肺腺癌细胞株进一步开展了TITF1/NKX2-1敲除实验，发现这两种细胞株的TITF1/NKX2-1均呈高表达。在NSCLC肿瘤中，34种潜在的TITF1/NKX2-1调节基因的蛋白质产物的表达与*TITF1*/*NKX2*-*1* mRNA水平密切相关。

在EGFR突变和KRAS突变肺腺癌小鼠模型以及SCLC小鼠模型中，生物反应路径分析（Ingenuity Pathway Analysis）发现了涉及EGFR的蛋白质网络，同时，对SCLC小鼠模型的血浆蛋白质组进行分析也发现了33种与神经疾病通路或神经系统进展及功能通路有关的蛋白质。

EGFR突变小鼠模型中的血浆EGFR网络包括六种直接与EGFR结合的蛋白质（Met、Cd44、Cdh1、Ndn、Sh3bgrl以及Rin1）。有趣的是，仅在EGFR突变小鼠模型中观察到Cd44（IPI00265503）的一种形态，而在KRAS小鼠模型中未发现。对21种肺腺癌细胞株的蛋白质组学分析发现，在10种EGFR突变细胞株中，其中4种可见编码CD44变异区的多肽，但在11种KRAS突变细胞株中均未观察到。

对比厄洛替尼治疗前后携带*EGFR*^*L858R*^转基因荷瘤小鼠的血浆蛋白质谱，发现了小鼠血清中表达改变的91种蛋白质的表达水平恢复到基线水平。利用这一方法，亦发现了EGFR模型中可反映肿瘤进展和回归的蛋白质。

两组NSCLC人血液样本（诊断前0-11个月以及新近诊断的吸烟者）、一组SCLC人血浆样本及其匹配的非肿瘤对照中，应用ELISA方法对小鼠模型中发现的蛋白质进行检测。在NSCLC患者中，EGFR水平降低，而SFTPB、WFDC2以及ANGPTL3水平升高。在拟诊断样本组，该四种蛋白质联合的曲线下面积（area under the curve, AUC）为0.808，新近诊断组的AUC为0.882。靶向于ROBO1外功能区的ELISA检测亦显示，与NSCLC患者及非肿瘤对照血浆中的水平相比，SCLC人血浆中ROBO1水平显著升高。人肺癌样本所得结果与来自小鼠模型的发现完全吻合。

## 讨论

蛋白质浓度是动态变化的，且有时会随着应激、疾病或治疗发生明显改变^[3]^。对有机体的蛋白质组进行检测就好像在某一时间点对其活动进行“快照”。这可能会低估或错失其余时间发生的过程的重要性。由于个体的异质性，Taguchi等选择基因工程小鼠模型对肺癌蛋白质组进行研究是非常明智的。事实上，他们的研究不仅广泛探索了不同组织类型肺癌模型的蛋白质组，而且通过可靠的方法进一步验证了一些已发现的重要蛋白质，这为蛋白质组生物标志物的发现及验证树立了新的榜样。

该研究采用了许多携带有不同肺癌类型以及不同肿瘤类型的小鼠模型。尽管如此，为强调肿瘤进展中的转化变化，应纳入良性和转移性肺癌肿瘤模型^[4]^。他们也应用了两种炎症小鼠模型，目前小鼠模型仍是探讨炎症在肺癌进展中作用的热点^[5]^。该研究所应用的急性、慢性炎症动物模型有助于我们理解癌症和炎症模型之间的区别。为对肺癌进展有更明确的了解，纳入肺相关炎性疾病（如：慢性阻塞性肺疾病）的小鼠模型从而对肺癌复杂的微环境进行更为彻底的研究或许更好^[6]^。

尽管每组小鼠血浆均受到三种最为丰富的蛋白质（白蛋白、IgG以及转铁蛋白）通过多重亲和去除系统-3的免疫清除，一些已证实与肺癌相关的蛋白质在肺腺癌小鼠模型中并未被发现。作者解释为质谱分析灵敏度不足或这些蛋白质在肿瘤进展早期并未明显升高，需要更敏感的平台以改善检测性能。血清淀粉样蛋白A作为另一广泛存在的肺癌潜在生物标志物在研究所用的所有小鼠模型中均有发现但未被量化，该蛋白质值得量化，因为它或许是肺癌的潜在生物标志物^[7]^。

为验证小鼠模型中所发现的蛋白质与人肺癌是否相关，该研究分析了自小鼠模型中发现的四种蛋白质的组合用以检测26例拟诊断和28例新近诊断的人NSCLCs的潜在价值。他们的发现不仅验证了小鼠与人肺癌血浆蛋白质变化的一致性，而且也提示这一组合的明显变化可作为肺癌早期诊断的微创NSCLC特异性生物标志物。大型前瞻性多中心试验或许有助于该组合标志物的临床应用。

## 专家评论及5年展望

早期诊断有助于改善肺癌患者的生存，因此发现早期诊断生物标志物非常有意义^[8]^。该研究结果显示对小鼠模型的蛋白质表达的定量对比分析将有助于研究拟诊断和新近诊断的人NSCLCs。事实上，定量对比蛋白质组学方法用途极广，可以揭示许多问题。例如，Wei等利用该方法在肺腺癌细胞中发现了用于多药耐药和放疗抵抗的蛋白质标志物^[9]^。他们共鉴定出四种表达上调的蛋白质（HSPB1、波形蛋白、cofilin-1和膜联蛋白A4），这些蛋白质可作为预测人肺腺癌对化疗和放疗反应的潜在生物标志物。

目前发现的大多数肿瘤标志物对个别癌症类型来说并非十分特异，这限制了它们在个别癌症筛查中的应用。该项研究在已定量血浆蛋白质中成功应用聚类分析以揭示不同癌症类型中多种模型的聚类。该方法或有助于克服生物标志物肿瘤类型特异性的挑战。

新近大量基因研究已经发现包括不同信号通路在内的癌症标志^[10]^。该研究分析了NSCLC及SCLC模型中的蛋白质网络。突变EGFR肺癌模型血浆中EGFR网络的发现或许揭示了驱动肺肿瘤进展的基因和通路，而SCLC模型中发现的蛋白酶体通路或可为SCLC提供潜在的神经内分泌靶标。事实上，研究已证实功能蛋白质组学是通路分析的可行方法。通过在肺癌中进行功能性磷酸化蛋白质组学分析，Sudhir等发现了Ras信号通路中的一些潜在靶标^[11]^，他们的研究发现了可由致癌性Ras调控的磷酸化过程、信号网络和分子功能，应有助于开拓我们对肺癌中Ras信号的认识。Remily-Wood等利用液相色谱法串联多级反应检测质谱分析法以及GeneGO MetaCore^TM^的通路地图以明确蛋白质之间的生物关系并阐明多元化分析的理念^[12]^，他们最终研发了定量分析数据库（Quantitative Assay Database）^[101]^，实践了癌症中信号通路和生物学过程的蛋白质组的定量分析。在不远的将来，国际联盟和更多开放存取的数据库将成为现实。肿瘤生物学的探索正在涌现。

动物模型为转化研究架起了桥梁^[13]^。该研究将小鼠模型中的发现与人体临床样本的验证整合，这或可加速潜在的生物标志物用于NSCLC早期诊断的临床实践的成功。在未来5年内，我们预测多种蛋白质生物标志物将被用于肿瘤早期诊断。经大规模前瞻性多中心验证后，蛋白质组学分析所支持的体外和体内数据将迈向临床实践^[14]^。

## Financial & competing interests disclosure

*The author has no relevant affiliations or financial involvement with any organization or entity with a financial interest in or financial conflict with the subject matter or materials discussed in the manuscript*. *This includes employment*, *consultancies*, *honoraria*, *stock ownership or options*, *expert testimony*, *grants or patents received or pending*, *or royalties*. *No writing assistance was utilized in the production of this manuscript*.

**关键发现**

•     在肺腺癌小鼠模型中，深入的定量蛋白质组学发现了Titf1/Nkx2-1血浆蛋白质组。

•     血浆蛋白质标志物可反映肺癌的分子水平不同的亚型并反映肺肿瘤生物学。

•     在EGF受体突变模型中发现了一涉及EGF受体的蛋白质网络，并且在小细胞肺癌模型中发现了一种不同的与神经内分泌进展相关的血浆标志物。

•     血浆蛋白质网络揭示了一些可驱动肿瘤进展的基因。

•     小鼠模型中发现的蛋白质标志物与人肺癌样本的ELISA结果一致。

•     对比四种肺癌小鼠模型与乳腺癌、胰腺癌、卵巢癌、结肠癌和前列腺癌模型及两种炎症模型的血浆蛋白质谱，在小鼠模型的血浆中共发现16种肺癌特异性蛋白质标志物。
